# Eco-sustainable chitosan–PVA biocomposites reinforced with recycled alum: preparation, characterization, and antimicrobial assessment

**DOI:** 10.1039/d5ra09668e

**Published:** 2026-01-26

**Authors:** Mahmoud A. Abdelkawy, Abdelbaset Shoker, Badr Isamil Badr, Yehia A.-G. Mahmoud

**Affiliations:** a Chemistry Department, Faculty of Science, Tanta University Tanta 31527 Egypt mahmoud.abdelkawy@science.tanta.edu.eg; b Botany and Microbiology Department, Faculty of Science, Tanta University Tanta 31527 Egypt

## Abstract

This study presents a sustainable approach to valorize aluminum can waste by converting it into recycled potassium alum (Recy. Al), used as a reinforcing and bioactive filler in chitosan/poly(vinyl alcohol) (CS/PVA) biocomposites. Recy. Al was synthesized through a green precipitation process and incorporated (5–50 wt%) into CS/PVA matrices *via* solution casting. FTIR and XRD confirmed successful alum integration without altering the matrix's semi-amorphous structure. Thermal analysis revealed enhanced stability, with 5% and 10% weight-loss temperatures (*T*_d5_, *T*_d10_) increasing from 188 °C and 239 °C for pristine CS/PVA to 223 °C and 240 °C for the 50 wt% composite, along with a higher char yield (38%). Mechanical strength and stiffness improved, reaching 21.1 ± 3.3 MPa and 501.3 ± 15.9 MPa at 30 wt% loading. SEM/EDS confirmed uniform alum dispersion and strong polymer-filler adhesion. Biologically, the CS/PVA–20 wt% composite showed potent antibacterial activity (MIC = 7.8 ± 1.0 µg mL^−1^; MCD ≈ 15 µg mL^−1^) against *E. coli* and *S. aureus*, while the CS/PVA–10 wt% sample exhibited the highest antifungal effect against *S. cerevisiae* and *A. niger*. Cytotoxicity tests confirmed good biocompatibility up to 20 wt% Recy. Al. These findings demonstrate that biocomposite films are thermally stable, antimicrobial, and eco-friendly materials for sustainable applications.

## Abstract 2

This study presents a sustainable approach to valorize aluminum can waste by converting it into recycled potassium alum (Recy. Al), used as a reinforcing and bioactive filler in chitosan/poly(vinyl alcohol) (CS/PVA) biocomposites.

## Graphical abstract

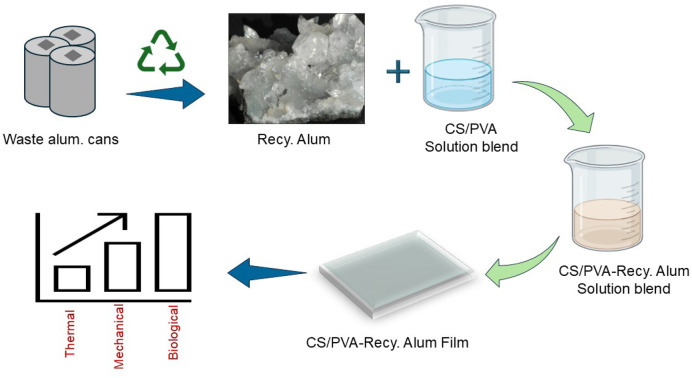

## 1. Introduction

The rapidly growing global population, combined with the fast pace of industrialization, has caused a significant increase in waste production across domestic, agricultural, and industrial sectors.^1^ These waste streams pose a significant threat to natural resources, public health, and environmental sustainability. Approximately 2 billion tonnes of municipal solid waste are generated annually, and by 2030, this is projected to grow to 2.59 billion tonnes.^2^ This burden is compounded by agricultural residues, such as crop straw and husks, and agro-industrial byproducts, like bagasse and fruit peels, especially in developing countries where open burning remains a common but environmentally harmful disposal method.^3,4^ An extra 20 million tonnes are added each year from industrial waste, which includes both hazardous and non-hazardous materials that leach into land and water ecosystems. This waste encompasses byproducts from mining, manufacturing, and chemical processing.^5,6^

Interest in converting waste into value-added materials has intensified in response to the global shift toward sustainability and waste valorization. For instance, lime sludge has been utilized in polymer composites, fly ash has been repurposed for low-carbon construction materials, and plastic waste has been chemically recycled through depolymerization.^7–9^ Among industrial byproducts, alum sludge generated during the coagulation–flocculation process of water treatment using aluminum-based coagulants has attracted increasing attention. However, its disposal by landfilling poses significant environmental and economic challenges due to the presence of organic matter, suspended solids, and aluminum hydroxide.^10^ Although alum sludge has been explored for applications in adsorbents,^11^ construction materials,^12^ and agriculture,^13,14^ its incorporation into polymer matrix composites remains largely unexplored.

Recent studies and reviews on aluminum waste valorization have mainly focused on recycling aluminum scrap into metal matrix composites (MMCs), where recycled aluminum acts as the matrix and ceramic fillers are used to improve mechanical and thermal performance for high-end structural applications.^15–17^ These approaches generally require high-temperature processing, strict impurity control, and energy-intensive routes, which limit their environmental sustainability and applicability in polymer-based systems. Moreover, most existing studies emphasize aluminum recovery and purification rather than its direct functional utilization as a reinforcing phase in polymer or biopolymer composites. In contrast, the incorporation of recycled aluminum into biodegradable polymer matrices remains largely unexplored, particularly for applications requiring low processing temperatures, antimicrobial functionality, and environmental compatibility. Addressing this gap, the present work proposes a sustainable upcycling strategy that directly integrates aluminum can waste into a chitosan/PVA biopolymer matrix, enabling simultaneous enhancement of mechanical performance and antimicrobial activity through a low-cost and environmentally benign route.

The development of “green polymers” made from renewable resources has accelerated in both academia and industry due to the need for waste valorization.^18^ Biodegradability, environmental friendliness, and lower energy requirements in production are among the obvious benefits of biopolymers over petroleum-based polymers.^19^ However, their broader adoption is often limited by intrinsic issues in their mechanical strength, thermal stability, and processability.^20–22^ Hybrid systems that combine natural and synthetic polymers are actively being researched as solutions to these challenges.

After cellulose, chitosan (CS), a linear polysaccharide derived from the partial deacetylation of chitin, is the second most common natural biopolymer. Its abundant reactive amino and hydroxyl groups provide beneficial qualities such as antimicrobial activity, biodegradability, biocompatibility, and non-toxicity.^23,24^ Due to these qualities, CS is a versatile option for applications in biomedical fields such as tissue engineering, drug delivery, wound healing, as well as food packaging. Polyvinyl alcohol (PVA), a synthetic water-soluble polymer with a hydroxyl-rich backbone that promotes extensive hydrogen bonding, is well known for its biodegradability, high crystallinity, and strong film-forming ability.^25^ Its mechanical strength, chemical stability, and compatibility with natural polymers make it suitable for packaging and biomedical uses. When CS and PVA are blended, their combined properties offer a sustainable way to produce functional composite films. However, issues like reduced flexibility and poor thermal stability can occur, especially with higher CS content. The stiff structure of CS limits polymer chain mobility, and studies have shown that increasing chitosan loading tends to lower the elongation at break and thermal degradation temperature of CS–PVA blends.^26–28^ Despite these drawbacks, CS/PVA composites remain promising for eco-friendly material development. To improve mechanical and thermal properties while maintaining biodegradability, approaches such as adding inorganic fillers or plasticizers have been explored.

The global push toward sustainable materials and waste valorization has spurred increased research into repurposing waste into functional materials. There is an urgent need for antimicrobial agents, antibiotics, and biocides (such as antiseptics, disinfectants, and preservatives) to control human pathogens. Many efforts are underway to develop new antibacterial, antifungal, antiviral, and anti-cancer drugs. It is estimated that around 700 000 people die each year due to infections with resistant bacteria, highlighting the urgent global need for new antimicrobial agents for current and future generations.^29^ The search for novel antimicrobial compounds from various sources is critical but challenging. Pharmaceutical companies must invest significant time and money into research, involving multiple rigorous phases of synthesis, discovery, and testing of new agents that must pass demanding criteria.^30^ Therefore, this study aims to develop biocomposites capable of combating microbial contamination, which is a major concern across sectors such as medical devices, healthcare products, water purification, hospitals, dental offices, food packaging, and storage. The study also evaluates both the cidal and static effects of the synthesized agents on test organisms. Additionally, our research focuses on creating a sustainable, high-performance biocomposite by incorporating recycled aluminum particles from cans into a chitosan/PVA matrix. This innovative approach addresses both aluminum waste management and the mechanical limitations of biopolymers while providing an eco-friendly pathway to value-added materials suitable for sustainable packaging, structural films, and environmental coatings. By harnessing the reinforcing properties of recycled aluminum particles and the synergistic interaction between CS and PVA, we aim to develop a biocomposite that is mechanically durable, thermally stable, and environmentally friendly.

## 2. Materials and methods

### 2.1. Materials

Poly(vinyl alcohol) (PVA) with a purity of (>98.0%), and Chitosan (CS) with a purity of (99.0%) (*M*_w_ = 100–300 kDa), with a 90% degree of deacetylation, were provided by Sigma-Aldrich. Glacial acetic acid (99%) was used without further purification from Adwic Co., Egypt.

### 2.2. Experimental methods

#### 2.2.1. Synthesis of recycled alum (Recy. Al)

In a previously published method, waste aluminum cans were first cleaned by mechanically removing the internal polymer coatings and external paint, and then they were cut into small pieces.^31^ A colorless potassium aluminate solution with the evolution of hydrogen gas was produced when the pieces were reacted with 25 mL of 3 M KOH solution in a beaker under a fume hood. The mixture was filtered to get rid of the insoluble residues after it had completely dissolved. After treating the clear filtrate with 3 M H_2_SO_4_, a white, gelatinous Al(OH)_3_ precipitate first formed. This precipitate dissolved when more acid was added, producing Al^3+^, K^+^, and SO_4_^2−^ ions. To allow potassium aluminum sulfate (alum) to crystallize, the solution was left undisturbed for the entire night. After filtering and washing them with a solution of ethanol and water, the crystals were left to dry overnight at room temperature. In line with earlier findings, the total recovery yield of the recycled alum from aluminum cans was roughly 80%. In subsequent analyses, commercial potassium alum (KAl(SO_4_)_2_·12H_2_O, ≥99% purity, Sigma-Aldrich) was used as a reference material for comparison. The recycled alum was then added to the chitosan/PVA (CS/PVA) biocomposite as a reinforcing agent.

#### 2.2.2. Preparation of CS/PVA-reinforced Recy. Al bio-composite films

**Fig. 1:**
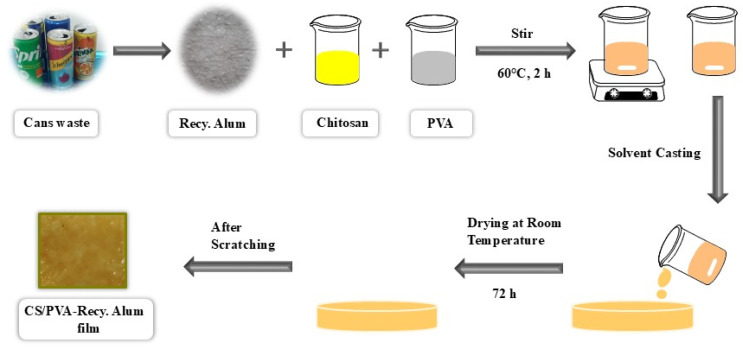
Schematic representation of prepared CS/PVA-Recy. Al films.

Fig. 1 illustrates the complete process for the preparation of CS/PVA–Recy. Al films. Solution casting was used to create biocomposite films. One gram of chitosan flakes was dissolved in a 2.0% (w/w) aqueous acetic acid solution while being constantly stirred for five hours at 60 °C. Parallel to this, PVA (0.5 g) was dissolved in deionized water for five hours at 80 °C while being constantly stirred. Before blending, both solutions were allowed to cool to room temperature. Recycled alum (Recy. Al) was incorporated into the CS/PVA blend at different loadings (5, 10, 20, 30, and 50 wt%). The required amount of Recy. Al powder was first dispersed in ultra-pure water and then added to the CS/PVA solution, followed by vigorous stirring for 30 min to ensure homogeneous dispersion before film casting. Following casting onto glass Petri dishes, the resultant mixtures were dried for five days at 60 °C. After being peeled from the dishes, the dried films were kept in a desiccator until they could be characterized.

### 2.3. Biological application

#### 2.3.1. Antimicrobial sensitivities test determination

Antimicrobial activity was evaluated by assessing the reduction in bacterial and fungal count. The activities of the produced compounds have been proved by estimation of inhibition zone diameters (mm) formed around 60 mg of compounds under study. The modified Kirby–Bauer disc diffusion technique was used to evaluate the antimicrobial sensitivity tests.^32^ The antimicrobial sensitivities had been done against Gram-negative bacteria *Escherichia coli* (*E. coli*) and *Proteus* sp., and Gram-positive bacteria *Staphylococcus aureus* (*S. aureus*), *Bacillus subtilis* (*B. subtilis*), and fungi *Saccharomyces cervisiae* (*S. cervisiae*) and *Aspergillus niger* (*A. niger*). Fungi and bacterial isolates were obtained for our culture collections (Botany Department, Tanta University, Egypt). Nutrient agar medium is used for the cultivation of all bacterial isolates (3 g beef extract, 5 g peptone, and 15 g agar per liter of medium) and nutrients broth (Nutrients broth (same formulation without addition of agar)). On the other hand, Sabouraud Dextrose Agar (20 g glucose, 10 g peptone, and 15 g agar per liter of medium) and Sabouraud Dextrose Broth (same formulation of Sabouraud Dextrose agar without addition of agar) were used to grow both fungal species. A loop full of bacterial and fungal growth was activated in Nutrient broth for bacteria and Sabouraud Dextrose broth for fungi. The inoculated culture broths were incubated at 37 °C for 24 h. In 9.0 mL Petri dishes, 100 µL of the test organisms' suspensions (A bacterial inoculum was 1.1 × 10^6^ CFU per mL, and fungal isolates inoculum was 1.2 × 10^5^ CFU per mL) had been added. After solidification of the media, 60 mg from different synthesized compounds were added. The Can wastes were also used as control. The inoculated Petri dishes are incubated at 37 °C for 24–48 h. The different inhibition zones (mm) were measured in triplicate (*n* = 3).

#### 2.3.2. Determination of minimum inhibitory concentrations (MICs) of synthesized biocomposites

Pristine CS/PVA, CS/PVA-5 wt% Recy. Al, CS/PVA-10 wt% Recy. Al, CS/PVA-20 wt% Recy. Al, CS/PVA-30 wt% Recy. Al, CS/PVA-30 wt% Recy. Al and CS/PVA-50 wt% Recy. Al. All were subjected to determine their minimum inhibitory concentration (MIC) using the broth serial dilution method.^33^ The MICs were determined against *E coli*, *S. aureus*, *S. cervisiae,* and *A. niger*. The tested microorganisms were grown in their proper nutrient broth medium as previously described. Dilutions of the biocomposites were dissolved in nutrient broth for bacteria and Sabouraud Dextrose broth for fungi. In a series of concentrations of biocomposites were diluted in test tubes containing 5 mL of the appropriate broth media to obtain the following concentrations: 500 µg mL^−1^, 250 µg mL^−1^, 125 µg mL^−1^, 62.50 µg mL^−1^, 31.25 µg mL^−1^, 15.625 µg mL^−1^, 7.8125 µg mL^−1^, and 0.0 µg mL^−1^. Afterwards, tubes containing nutrient broth or Sabouraud Dextrose medium broths were inoculated with microbial suspensions (for bacteria 1.2 × 10^6^ CFU mL^−1^ and for fungi 2 × 10^5^ CFU per mL) after initiation, then agar plates were inoculated with 100 µL per culture, well spread, and the plates were incubated at 37 °C for 24–48 h according to the type of microorganism. The number of colonies was counted as CFU per mL. The assay was performed in triplicate (*n* = 3).

#### 2.3.3. Cidal and/or static effects of a new synthetic biocomposite by death/MICs index

This experiment aimed to examine the cidal and/or static effects of a new synthetic biocomposite. In a series of concentrations of biocomposites diluted in test tubes containing 5 mL of the appropriate broth media contained the following concentrations: 500 µg mL^−1^, 250 µg mL^−1^, 125 µg mL^−1^, 62.50 µg mL^−1^, 31.25 µg mL^−1^, 15.625 µg mL^−1^, 7.8125 µg mL^−1^ and 0.0 µg mL^−1^ (control) in nutrients broth or Sabouraud Dextrose broths. The minimum effective concentration of complete death (MCD) of an antibacterial or antifungal agent was defined as the concentration at which all bacteria or fungi were eradicated. On the other hand, concentration was found to prevent observable bacterial growth in the MICs.^34^ The minimum effective concentration of complete death (MCD) was examined as MICs. The agar plates were incubated at 37 °C for 24–48 h. To choose the MCD value, since it was the lowest concentration on the agar plates that did not produce any discernible growth or all microorganisms were eradicated completely using CFU per mL. Triplicates of each experiment were carried out.^35^ The MCD/MIC ratio can determine if the bacteria are susceptible, tolerant, or resistant to a certain agent that is being challenged. A compound was considered a bactericidal or fungicidal agent if the tolerance value (MBC/MIC) was ≤4, and bacteriostatic or fungistatic if the tolerance value (MBC/MIC) was >4.^36^

#### 2.3.4. The cytotoxicity test of synthesized biocomposites (mg mL^−1^) using artemia in seawater

The cytotoxicity or lethality effects on the brine shrimp (*Artemia salina*) of biocomposites have been studied. The examined biocomposites are (Pristine CS/PVA, CS/PVA-5 wt% Recy. Al, CS/PVA-10 wt% Recy. Al, CS/PVA-20 wt% Recy. Al, CS/PVA-30 wt% Recy. Al, CS/PVA-30 wt% Recy. Al, and CS/PVA-50 wt% Recy. Al). The cytotoxicity or lethality effects of every synthesis of biocomposites were studied using the following concentrations (0, 10, 100, and 1000) mg mL^−1^. Cytogenic biocomposites caused the brine shrimp's high lethality wide use in health care. The survival ratio of artemia was calculated, and the LD_50_ was estimated.^37^

#### 2.3.5. Statistical analyses

The statistical analyses were done using SPSS 11. To test the significance and standard deviations, a *T*-test was calculated. The figures were drawn by Microsoft Office 2010.

### 2.4. Characterization

#### 2.4.1. FTIR analysis

The Fourier transform infrared spectroscopy (FTIR) analysis of the sample films was performed using a JASCO FT-IR 210 spectrometer. The analysis was carried out in the range from 4000 to 400 cm^−1^ with a 4 cm^−1^ resolution and a total of 32 scans. The FTIR spectra were recorded in transmittance mode.

#### 2.4.2. XRD analysis

The X-ray diffraction (XRD) was performed using a SmartLab (Rigaku Denki Co. Ltd). CuKα X-ray (*λ* = 1.5418 Å) beams that were monochromatized with a confocal mirror were irradiated onto the specimen through a pinhole collimator with a diameter of 0.2 mm. The working voltage and current were 40 kV and 20 mA, respectively.

#### 2.4.3. Thermal analysis

Thermal properties of the products were evaluated *via* thermogravimetric analysis (TGA) using a Seiko TGA6200 analyzer and differential scanning calorimetry (DSC) on a Seiko EXSTER DSC 6200 instrument at a heating rate of 10 °C min^−1^ under a nitrogen atmosphere. The samples were heated at the rate of 10 °C min^−1^ from 35 to 700 °C.

#### 2.4.4. Mechanical properties

The mechanical properties of the CS/PVA-Recy. Al biocomposites and neat CS/PVA were examined. Tensile tests were performed using a MX2-500N (IMADA Co., Ltd) with a digital force gauge ZTA-50N machine (IMADA Co., Ltd). Each test was repeated at least three times. Dogbone-shaped specimens were cut from 1 mm sheets, and three replicates were tested for each formulation to calculate average values and standard deviations. The results are reported as mean values with standard deviations to reflect measurement variability.

#### 2.4.5. Surface morphology

The surface morphology of the sample films was evaluated using scanning electron microscopy (SEM), A Hitachi SU8220 (Tokyo, Japan) was used with an operating voltage of 1.0 kV at a magnification of 20 000× at room temperature. Each sample was put on a holder before being coated with a thin platinum layer to avoid the charging effect.

#### 2.4.6. Moisture content and water absorption

The moisture content of the bio-composite films were determined by oven drying at 105 °C for 24 h until constant weight, followed by cooling in a desiccator prior to weighing. Water absorption behavior was evaluated following ASTM D570 by immersing dried specimens in distilled water at room temperature. The percentage of water uptake was calculated as a function of immersion time. The results represent the average of three measurements.
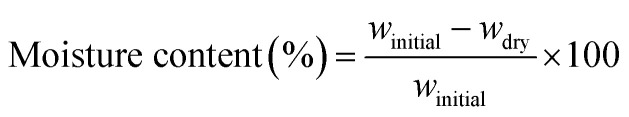

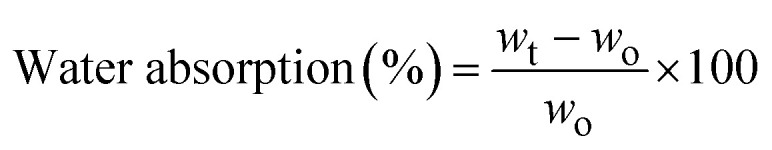


## 3. Results and discussion

### 3.1. Characterization of recycled potassium alum (Recy. Al)

Potassium alum (KAl(SO_4_)_2_·12H_2_O) is a widely accessible, inexpensive, water-soluble, and environmentally safe compound that is frequently used in laboratory and industrial settings without the need for special handling.^38–40^ In this study, the successful synthesis of potassium alum from recycled aluminum can waste using a green precipitation method produced a recovery efficiency of approximately 80%, which is consistent with earlier reports on alum regeneration from waste sources. FTIR and XRD analyses of the structural characteristics of the recovered alum verified that the recycled material had a high level of purity and crystallinity, comparable to that of commercial potassium alum.

**Fig. 2:**
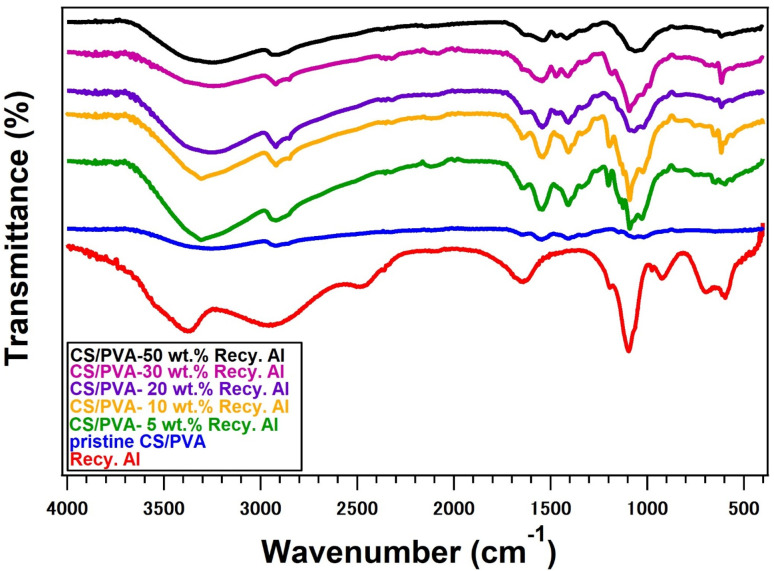
FTIR of pure Recy. Al, and 5–50 wt% Recy. Al-CS/PVA biocomposites.

The FTIR spectrum of the recycled alum exhibited several characteristic peaks (Fig. 2), confirming the successful formation of hydrated sulfate salt. Strong absorption bands at approximately 1191 cm^−1^ and 1095 cm^−1^ are attributed to the asymmetric stretching of the S=O groups. Additional peaks at 919 cm^−1^ and 698 cm^−1^ correspond to the stretching vibrations of the S–O and Al–O bonds, respectively. In the low-frequency region (750–400 cm^−1^), multiple bands appeared due to the Al–O lattice vibrations. Moreover, distinctive sulfate ion (SO_4_^2−^) bands were observed in the ranges of 449–480 cm^−1^, 595–609 cm^−1^, 657–698 cm^−1^, 1095–1110 cm^−1^, and 1291–1207 cm^−1^, which are consistent with the symmetric and asymmetric bending and stretching modes of the sulfate tetrahedron.^41,42^

X-ray diffraction (XRD) analysis further confirmed the crystalline nature of the recycled alum, exhibiting a diffraction pattern closely aligned with that of standard potassium alum (KAl(SO_4_)_2_·12H_2_O). The recycled alum displayed distinct and well-resolved diffraction peaks at 2*θ* values of 12.6°, 16.2°, 17.8°, 20.5°, 21.9°, 26.4°, 27.3°, 29.2°, 30.2°, 31.1°, 32.0°, 33.8°, 34.6°, 36.1°, 39.9°, and 40.6°, as shown in Fig. 4. The high crystallinity and lack of detectable impurities in the recycled alum were confirmed by the distinct and sharp diffraction peaks. This confirms the effectiveness and dependability of the upcycling process by showing that its structural characteristics closely resemble those of potassium alum made commercially.^41,43^

### 3.2. FTIR analysis of bio-composites

**Fig. 3:**
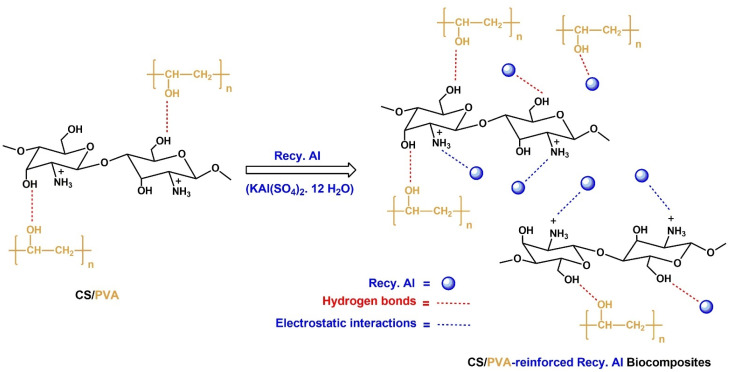
Possible interaction in CS/PVA and CS/PVA-reinforced Recy. Al biocomposite films.

Fig. 2 presents the FTIR spectra of the pristine CS/PVA blend and CS/PVA biocomposites reinforced with different loadings (5, 10, 20, 30, and 50 wt%) of recycled alum. The pristine CS/PVA film exhibited a broad absorption band in the region of 3200–3400 cm^−1^, arising from overlapping O–H and N–H stretching vibrations. This broadening indicates the formation of strong intermolecular hydrogen bonding between the hydroxyl groups of PVA and the hydroxyl and amino groups of chitosan, confirming good miscibility within the blended polymer matrix (Fig. 3).^43^ The absorption band near 2917 cm^−1^ corresponds to C–H stretching vibrations, while the band at approximately 1558 cm^−1^ is attributed to N–H bending vibrations. Additional bands in the region of 1000–1100 cm^−1^ are associated with the C–O stretching vibrations of the polymer backbone.^44–46^

The incorporation of recycled alum into the CS/PVA matrix resulted in noticeable changes in the FTIR spectra as a function of the filler content. The broad O–H/N–H stretching band at approximately 3357 cm^−1^ becomes slightly reduced in intensity and broadens with increasing alum loading, indicating enhanced hydrogen bonding interactions between the polymer functional groups and the alum surface. Moreover, the appearance and gradual intensification of absorption bands in the range of 1081–1190 cm^−1^ can be attributed to the overlapping of polymer C–O vibrations with the asymmetric stretching modes of sulfate (S=O) groups originating from the recycled alum. In the CS/PVA-Recy. Al composites, this band is shifted to 1540 cm^−1^. The characteristic band at 1200–1110 cm^−1^ indicates that the protonated amino groups (–NH_3_^+^) of chitosan and the sulfate (–OSO_3_^−^) moieties of the recy. Al are forming strong intermolecular interactions.

Additional bands appearing at approximately 919 cm^−1^ and 698 cm^−1^ are assigned to S–O and Al–O stretching vibrations, respectively, confirming the successful incorporation of alum particles into the CS/PVA matrix. In the low-frequency region (750–400 cm^−1^), multiple bands are associated with Al–O lattice vibrations and sulfate ion bending modes (Fig. 3). These spectral features became more pronounced as the recycled alum content increased from 5 to 50 wt% further evidencing effective filler incorporation.

Importantly, no new absorption bands corresponding to covalent bond formation were detected, indicating that the interaction between the CS/PVA matrix and recycled alum was governed primarily by non-covalent interactions. The observed spectral shifts and intensity variations confirm the presence of hydrogen bonding and electrostatic interactions between the hydroxyl and amino groups of the polymers and sulfate/aluminum species of recycled alum. These interactions facilitated good interfacial compatibility and contributed to the reinforcing effect of the recycled alum within the CS/PVA biocomposites (Fig. 3).

### 3.3. X-ray diffraction (XRD) analysis of bio-composites

**Fig. 4:**
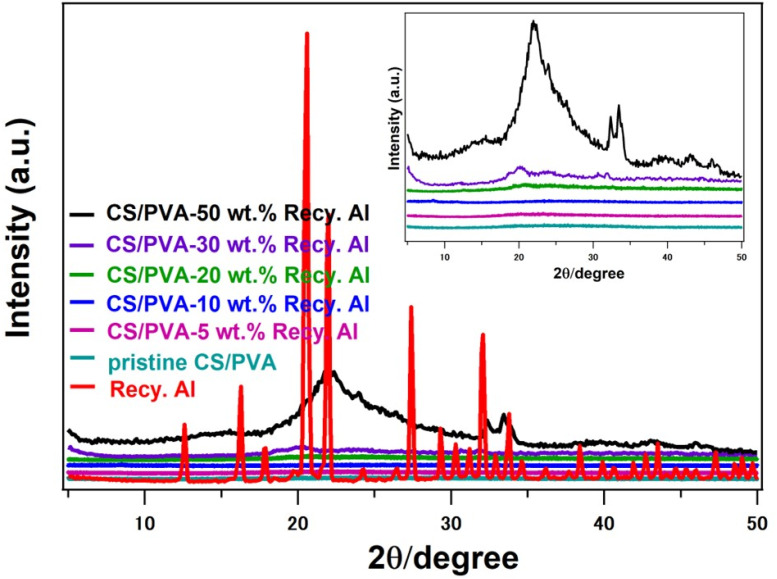
XRD traces of pure Recy. Al, and 5–50 wt% Recy. Al-CS/PVA biocomposites.

Fig. 4 shows the X-ray diffraction patterns of pristine chitosan–polyvinyl alcohol (CS/PVA) and the corresponding biocomposites containing recycled potassium alum at different loadings (5–50 wt%). In line with earlier reports on conventionally synthesized potassium alum, the recycled alum showed a distinct crystalline profile with multiple sharp reflections, indicating its high crystalline and structural purity. The pristine CS/PVA film, on the other hand, shows a wide, diffuse halo centered at 2*θ* ≈ 25°, which is consistent with the literature describing hydrogen-bonded CS/PVA networks and a feature of its primarily amorphous nature. The broad amorphous halo still dominates the polymer matrix diffraction pattern when recycled alum is added at low to moderate loadings (5–20 wt%). This suggests that alum is evenly distributed and does not create new crystalline phases or long-range ordering in the polymer backbone. Subtle but significant structural changes became apparent at higher loadings (≥30 wt%). At approximately 2*θ* ≈ 19°, 24°, 29.7°, 30.9°, and 31.6°, distinct crystalline reflections appeared along with a progressive narrowing of the main diffraction halo. These peaks represent the intrinsic crystalline domains of alum integrated into the matrix. Their appearance suggests localized ordering in the composite, which results from the physical confinement of the CS/PVA matrix remained predominantly amorphous due to restricted polymer chain mobility around alum aggregates.^47–49^ Although they encourage partial segmental alignment, these interfacial interactions, mostly hydrogen bonding and ionic associations, which remains predominantly amorphous.

### 3.4. Thermal analysis of bio-composites

**Fig. 5:**
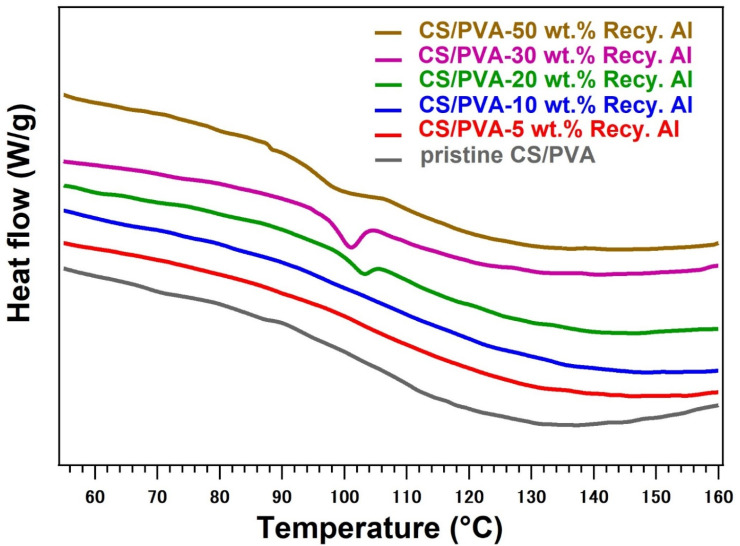
DSC thermograms of pristine CS/PVA, and 5–50 wt% Recy. Al-CS/PVA biocomposites.

**Fig. 6:**
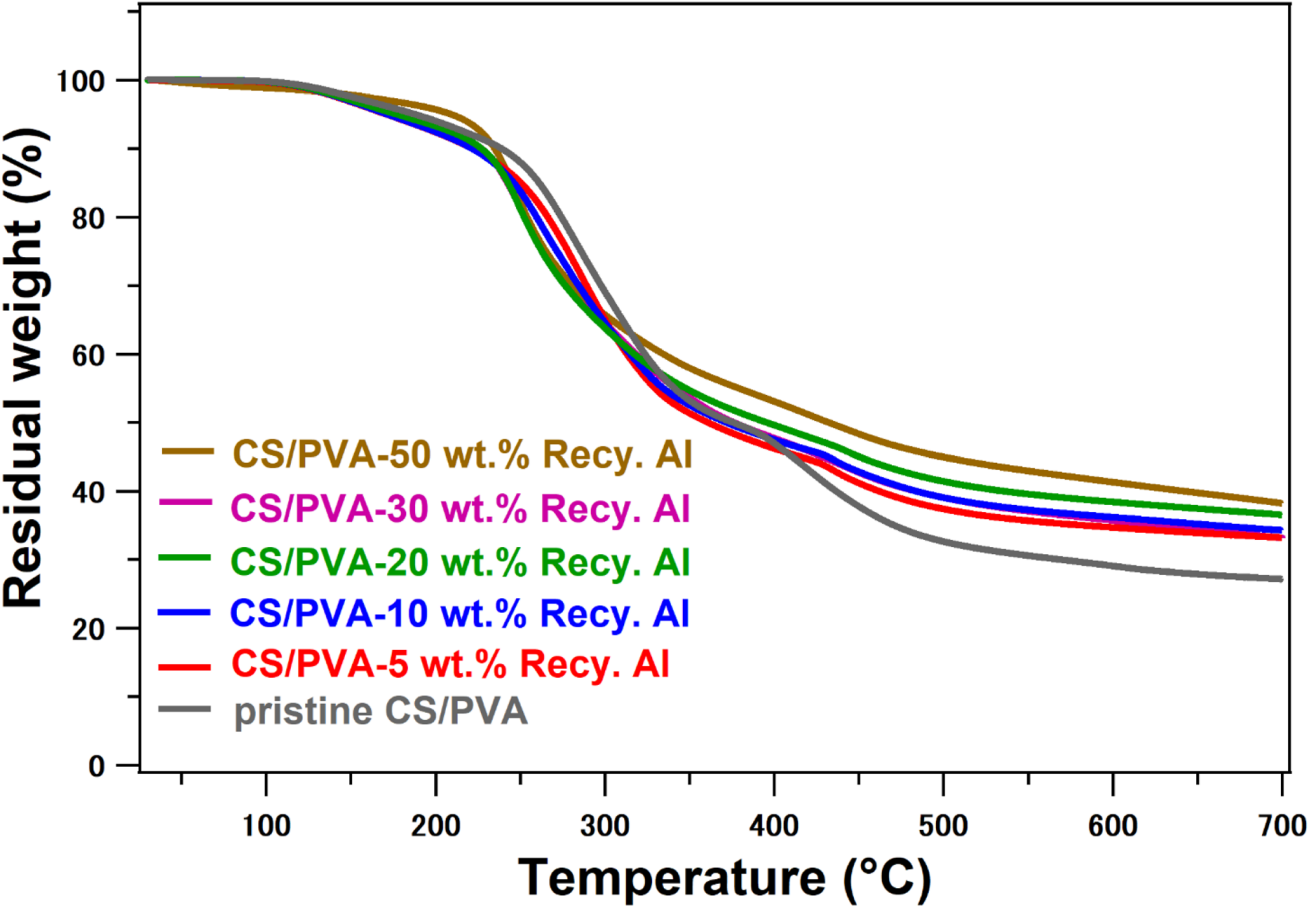
TGA profile of pristine CS/PVA and 5–50 wt% Recy. Al-CS/PVA biocomposites.

The thermal properties of pristine CS/PVA and its biocomposites reinforced with varying contents (5–50 wt%) of recycled alum were thoroughly investigated using Differential Scanning Calorimetry (DSC) and Thermogravimetric Analysis (TGA) under a nitrogen atmosphere. The results (Fig. 5, 6 and Table 1) reveal critical insights into the influence of Recy. Al on thermal transitions, decomposition behavior, and residual stability of hybrid materials.

**Table 1:** Thermal properties of pristine CS/PVA and CS/PVA-containing different loadings (5–50 wt%) of Recy. Al

Sample	*T*_g_ a (°C)	*T*_d5_ b (°C)	*T*_d10_ c (°C)	*Y*_c_ d (%)
Pristine CS/PVA	—	188	239	27
CS/PVA-5 wt% Recy. Al	—	168	220	33
CS/PVA-10 wt% Recy. Al	—	172	222	34
CS/PVA-20 wt% Recy. Al	102.7	176	228	36
CS/PVA-30 wt% Recy. Al	98.9	185	232	33
CS/PVA-50 wt% Recy. Al	96	223	240	38

a Glass transition temperature under nitrogen.

b Temperature for 5% weight loss under nitrogen in TGA.

c Temperature for 10% weight loss under nitrogen in TGA.

d Residual weight of the sample heated at 10 °C min^−1^ until 700 °C under nitrogen in TGA.

The DSC thermograms (Fig. 5) for the pristine CS/PVA blend did not exhibit a clearly defined glass transition within the measured range, which is consistent with its semi-amorphous hydrogen-bonded structure. Upon the incorporation of recycled alum, distinct endothermic relaxation events emerged, reflecting an increase in the microheterogeneity within the composite. At 20 wt% Recy. Al, a *T*_g_-like transition appeared at 102.7 °C, which decreased to 98.9 °C and 96 °C with further increases to 30 wt% and 50 wt% Recy. Al, respectively. This gradual downward shift is attributed to the disruption of polymer–polymer hydrogen bonding by the growing inorganic phase and the formation of alum–polymer interfacial regions in the gel. The presence of these microdomains weakens the segmental rigidity and promotes easier molecular relaxation, which is consistent with the localized structural ordering observed in the XRD patterns at higher alum loadings.^50^

The TGA results (Fig. 6) revealed the typical multistep degradation behavior of the CS/PVA systems. The initial mass loss below ∼120 °C corresponds to the evaporation of physically bound moisture and traces of acetic acid in the sample.^49,51^ Subsequent decomposition between 200 and 300 °C is associated with the cleavage of chitosan glycosidic linkages and the breakdown of PVA hydroxyl-bearing segments, while the third stage (300–550 °C) represents the degradation of intermediate residues and carbonaceous fragments, respectively.^52–54^

Pristine CS/PVA exhibited *T*_d5_ = 188 °C and *T*_d10_ = 239 °C, with a char yield of 27%. The addition of 5–20 wt% alum led to a slight decrease in *T*_d5_ (168–176 °C) and *T*_d10_ (220–228 °C), likely due to the catalytic dehydration effect of alum's sulfate content, which promotes early weight loss. Nevertheless, these composites showed a marked improvement in char yield, increasing to 33–36%, indicating that alum enhances residue formation and interacts favorably with the developing carbonaceous structure during decomposition. As the alum loading increased to 30 wt%, *T*_d5_ and *T*_d10_ shifted upward to 185 °C and 232 °C, respectively, suggesting reduced catalytic degradation owing to improved filler dispersion and more effective polymer–filler interfacial interactions. At the highest loading of 50 wt% Recy. Al, the biocomposite showed the most pronounced thermal enhancement, with *T*_d5_ = 223 °C, *T*_d10_ = 240 °C, and a char yield of 38%. This significant improvement is attributed to the dense inorganic network formed by alum, which acts as a thermal barrier, slowing heat transfer and restricting the diffusion of volatile degradation products. The high char yield further supports the formation of a more compact, ceramic-like protective layer at elevated temperatures.

### 3.5. Mechanical analysis of bio-composites

The mechanical behavior of polymer composites is strongly influenced by the dispersion quality and interfacial interactions of the filler within the matrix. Well-distributed inorganic particles can effectively hinder crack propagation, redistribute stress, and enhance load transfer efficiency, resulting in improved stiffness and tensile resistance.^50,55^ In contrast, excessive filler content often leads to particle agglomeration, microstructural discontinuities, and stress localization, ultimately diminishing ductility and compromising overall mechanical robustness.^56–59^

**Fig. 7:**
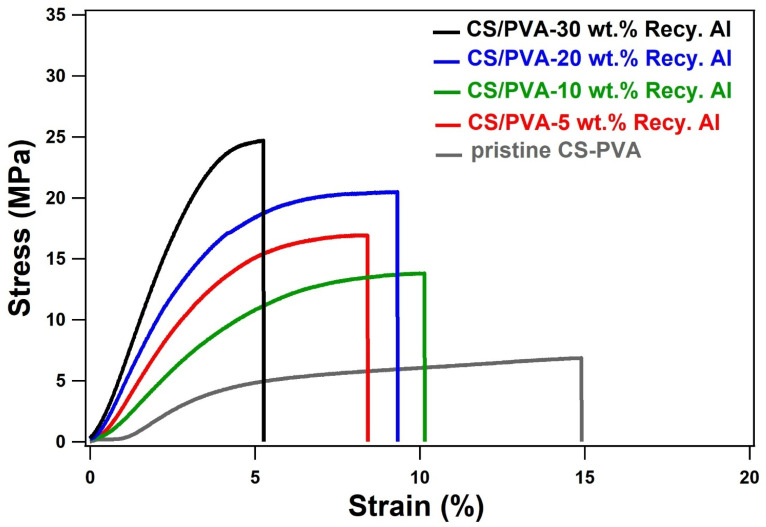
Stress–strain analysis for various combinations of pristine CS/PVA and CS/PVA-containing different loading (5–30 wt%) of Recy. Al.

The tensile data for pristine CS/PVA and the bio-composites containing 5–50 wt% recycled alum are presented in Table 2 and illustrated in the stress–strain curves of Fig. 7. The pristine CS/PVA film exhibited a tensile strength of 12.2 ± 6.4 MPa, Young's modulus of 84.2 ± 64.1 MPa, and elongation at break of 10.3 ± 4.2%, reflecting the characteristic flexibility and low intrinsic stiffness of the neat biopolymer matrix. Incorporation of 5 wt% recycled alum produced a notable strengthening effect, increasing tensile strength to 16.3 ± 1.3 MPa and stiffness to 129.2 ± 90.4 MPa, while the elongation improved to 15.4 ± 8.6%. This simultaneous enhancement in strength, modulus, and ductility suggests excellent particle dispersion and favorable polymer–filler interactions that facilitate efficient stress transfer without severely restricting chain mobility. At 10 wt% loading, the tensile strength remained high (16.7 ± 4.9 MPa) and the modulus increased further to 180.2 ± 78.6 MPa; however, the elongation decreased to 9.9 ± 4.6%. This reduction in flexibility is likely associated with the onset of localized heterogeneity or the formation of microaggregates that begin to restrict polymer relaxation. The 20 wt% composite exhibited the highest tensile strength among the tested films (19.0 ± 1.7 MPa) and a marked increase in stiffness (243.6 ± 32.6 MPa), confirming the reinforcing capability of alum at moderate loading. Nevertheless, the elongation dropped to 8.3 ± 1.8%, indicating the expected trade-off between stiffness and ductility as the inorganic phase became more dominant. Beyond this optimum range, the mechanical response changes. The 30 wt% film could not be reliably tested due to structural irregularities and premature failure, reflecting excessive filler aggregation and poor stress distribution. At 50 wt% loading, the composite exhibited extremely limited extensibility (4.6 ± 0.5%) and a significantly elevated modulus (501.3 ± 15.9 MPa), demonstrating that the matrix becomes highly rigid and brittle when the inorganic phase overwhelms the polymer network. Although the strength at this level (21.1 ± 3.3 MPa) appears comparatively high, the severe loss of elasticity and predominance of brittle fracture behavior significantly limit its practical applicability. These findings underscore the importance of balancing filler content to optimize mechanical performance and support the use of upcycled alum in developing cost-effective, environmentally friendly biocomposites suitable for packaging, coating, and structural applications.

**Table 2:** Mechanical properties of pristine CS/PVA and CS/PVA-containing different loading (5–50 wt%) of Recy. Al

Sample	*ε*_max_ a (%)	*σ*_max_ b (MPa)	Young's modulus^c^ (MPa)
Pristine CS/PVA	10.3 ± 4.2	12.2 ± 6.4	84.2 ± 64.1
CS/PVA-5 wt% Recy. Al	15.4 ± 8.6	16.3 ± 1.3	129.2 ± 90.4
CS/PVA-10 wt% Recy. Al	9.9 ± 4.6	16.7 ± 4.9	180.2 ± 78.6
CS/PVA-20 wt% Recy. Al	8.3 ± 1.8	19.0 ± 1.7	243.6 ± 32.6
CS/PVA-30 wt% Recy. Al	4.6 ± 0.5	21.1 ± 3.3	501.3 ± 15.9
CS/PVA-50 wt% Recy. Al	—	—	—

a Ultimate extensibility.

b Ultimate tensile stress.

c Young's modulus, calculated from the slope of flexural stress–strain curves.

### 3.6. Surface morphology of bio-composites

**Fig. 8:**
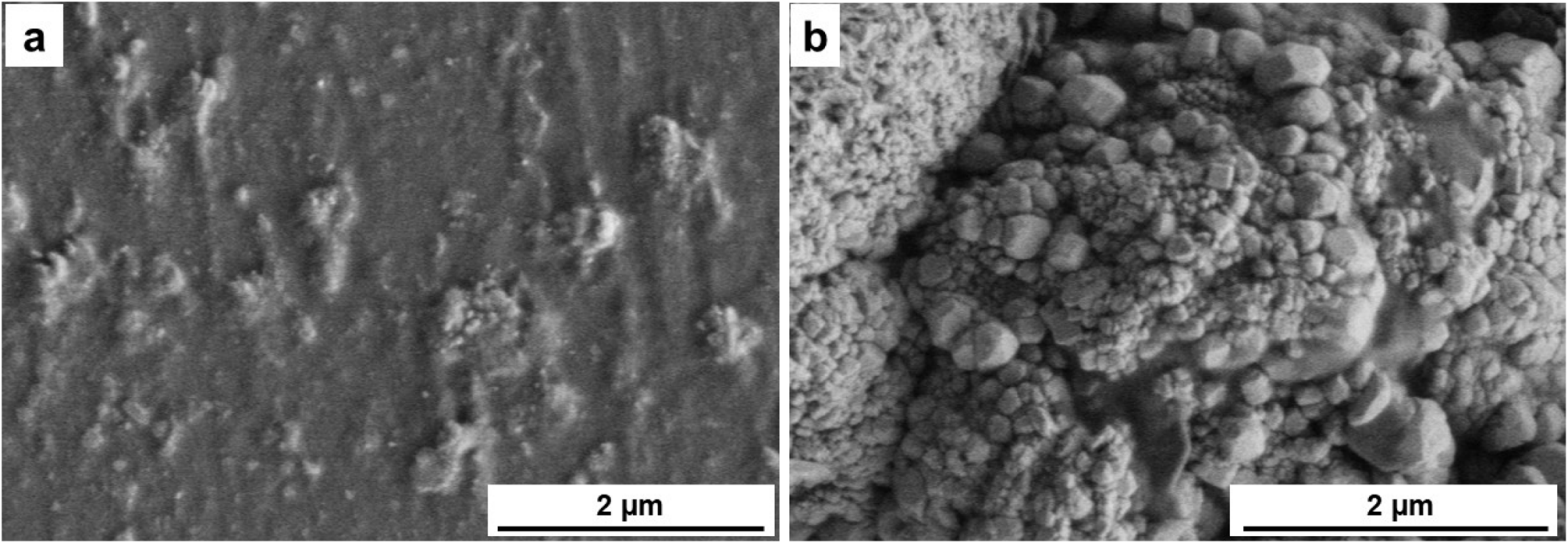
SEM images of (a) pristine CS/PVA and (b) CS/PVA-20 wt% Recy. Al.

The SEM micrographs (Fig. 8a) of the pristine CS/PVA film reveal a smooth, continuous, and homogeneous surface, confirming the excellent miscibility between chitosan and PVA. This uniformity is attributed to strong hydrogen bonding interactions between the hydroxyl and amino functionalities of both polymers, which promote the formation of a cohesive and well-integrated matrix.^44,45^ In contrast, the surface of the CS/PVA composite containing 20 wt% Recy. Al (Fig. 8b) displayed a rougher texture with distinct elongated, oval-shaped alum particles embedded within the matrix.^41,60^ The compatibility of recycled alum with the CS/PVA matrix was further facilitated by hydrogen bonding between the polymer functional groups (–OH and –NH_2_) and the alum surface. These features suggest particle agglomeration and partial alignment during film casting, likely because of the high filler concentration. The observed morphology is consistent with earlier reports on high inorganic filler content in polymer blends.

**Fig. 9:**
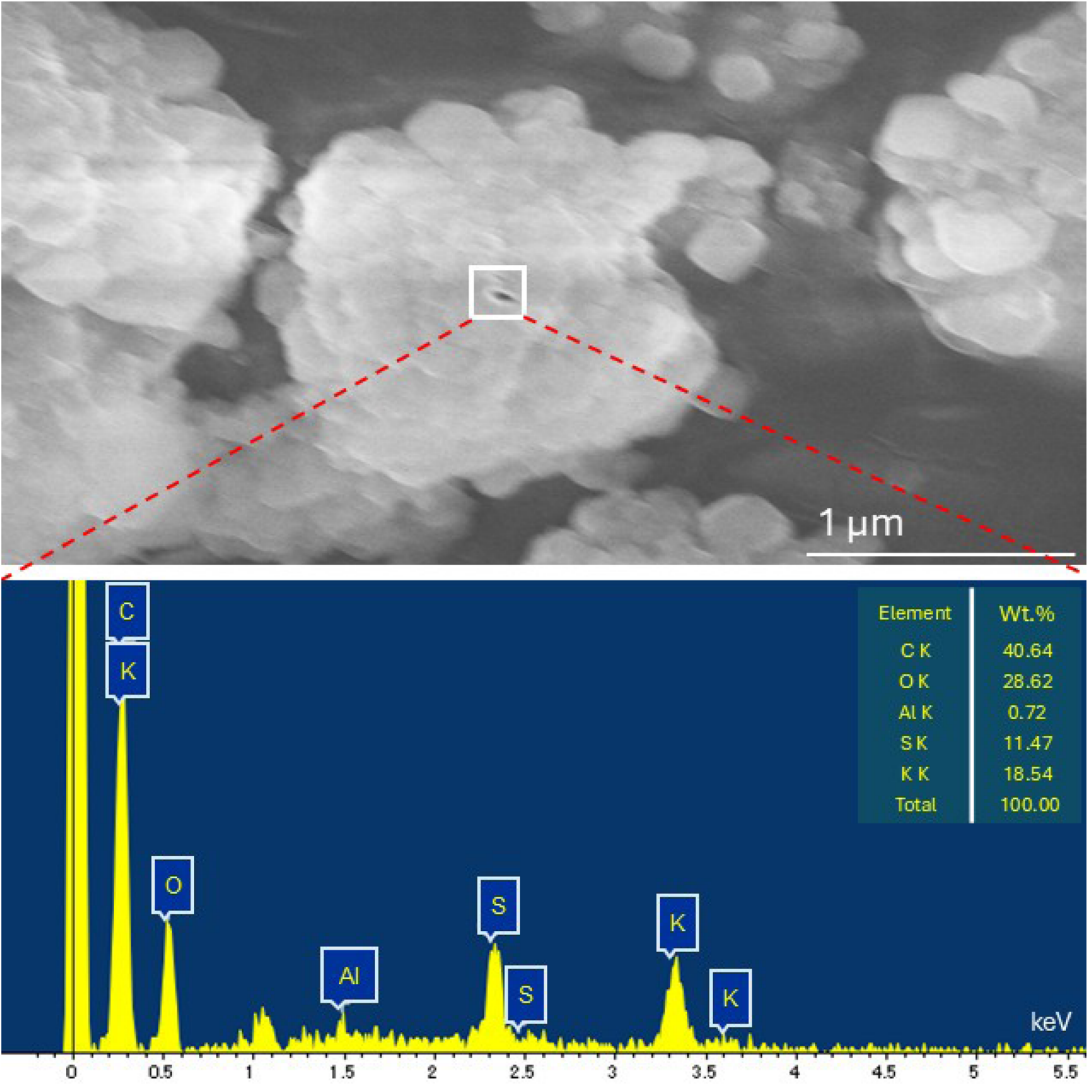
SEM-EDS analysis for the occurrence of Recy. Al in the fracture surface of 20 wt% Recy. Al-CS/PVA biocomposites.

**Fig. 10:**
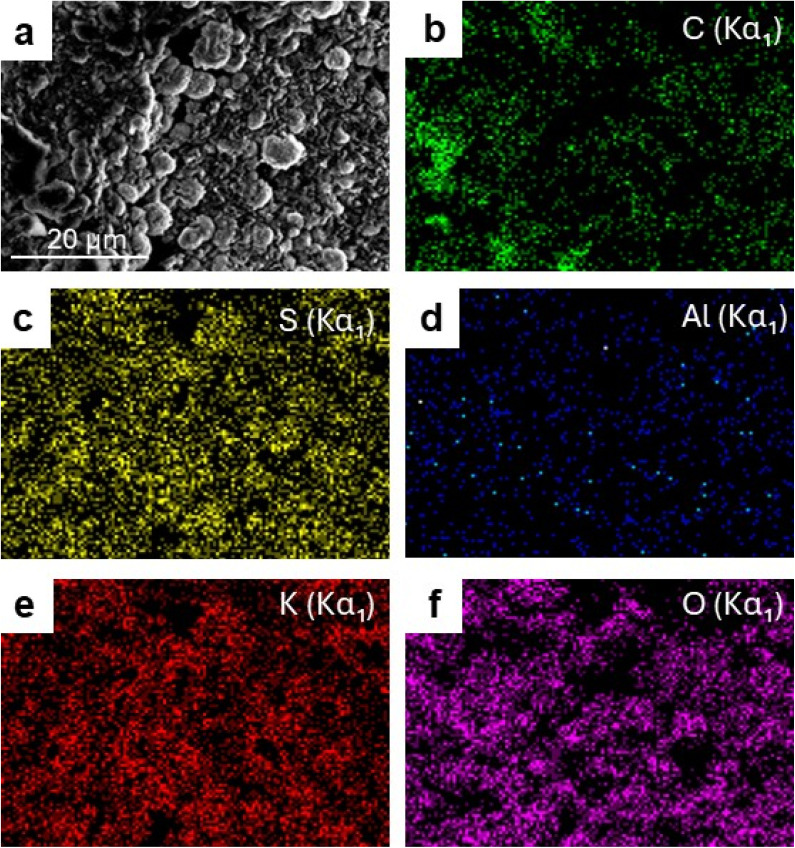
SEM image and corresponding EDS elemental mapping of the fracture surface of CS/PVA–30 wt% Recy. Al biocomposite: (a) SEM micrograph, (b) carbon (C), (c) sulfur (S), (d) aluminum (Al), (e) potassium (K), and (f) oxygen (O) elemental distribution maps.

The SEM–EDS spectrum (Fig. 9) further confirms the presence of recycled alum within the composite by detecting the characteristic elemental signals. Complementary elemental mapping analyses (Fig. 10a–f) illustrate the distribution of aluminum, sulfur, and oxygen across the fractured surface, verifying the successful incorporation of recycled alum and its interfacial embedding within the CS/PVA network. The relatively uniform elemental distribution, particularly at 30 wt% loading, indicates that the filler is effectively integrated, even though localized aggregation occurs at higher concentrations. These results confirm that recycled alum acts as a structurally influential filler in the CS/PVA matrix.

### 3.7. Moisture and water solubility of bio-composites

Moisture content and water solubility are critical parameters governing the dimensional stability, durability, and environmental performance of biopolymer-based composite films.^61^ Both chitosan (CS) and poly(vinyl alcohol) (PVA) are inherently hydrophilic due to the presence of abundant hydroxyl (–OH) and amino (–NH_2_) functional groups, which readily interact with water molecules through hydrogen bonding.

**Fig. 11:**
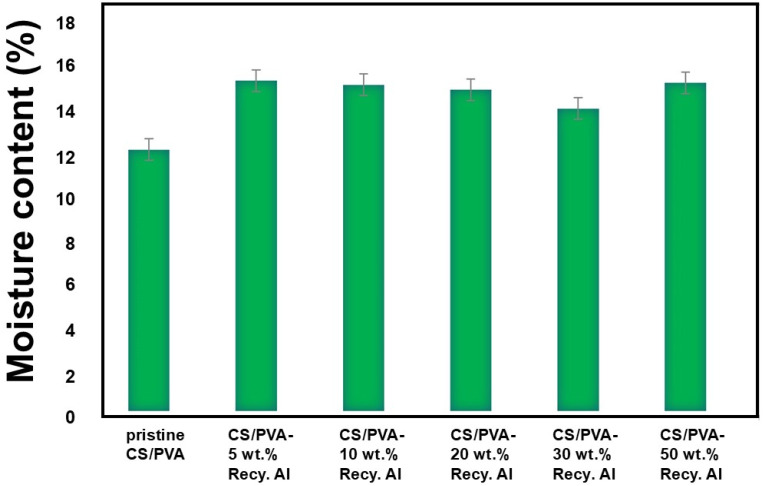
Moisture content of CS/PVA- Recy. Al bio-composites films.

The moisture content of pristine CS/PVA and CS/PVA–Recy. Al biocomposite films is presented in Fig. 11. The pristine CS/PVA film exhibited a moisture content of 13.6%, reflecting the strong affinity of the polymer blend toward atmospheric moisture. Upon incorporation of recycled alum, the moisture content increased to 17.18% and 16.97% for composites containing 5 wt% and 10 wt% Recy. Al, respectively. This increase can be attributed to the hydrated nature of potassium alum and the presence of sulfate groups, which enhance moisture retention through additional polar sites. With further increases in recycled alum content, the moisture content slightly decreased to 16.71% and 15.73% for 20 wt% and 30 wt% Recy. Al, respectively, suggesting improved matrix densification and reduced availability of free hydrophilic sites. At the highest filler loading (50 wt% Recy. Al), the moisture content increased again to 17.07%, likely due to the dominance of alum-associated hydration over polymer–polymer interactions.

**Fig. 12:**
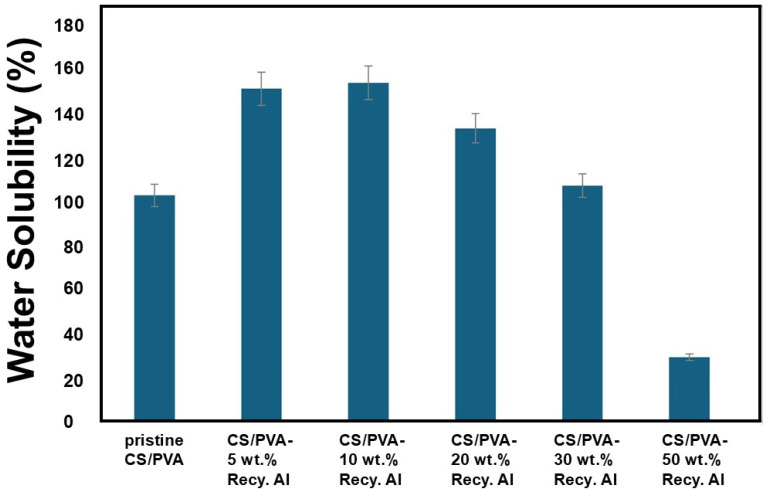
Water solubility of CS/PVA- Recy. Al bio-composites films.

The water solubility results of pristine CS/PVA and CS/PVA–Recy. Al biocomposites after 24 h immersion in distilled water are illustrated in Fig. 12. The pristine CS/PVA film exhibited a high water solubility of 114%, consistent with its hydrophilic nature. The incorporation of recycled alum at low loadings resulted in a pronounced increase in water solubility, reaching 168% and 171% for 5 wt% and 10 wt% Recy. Al, respectively. This enhancement is attributed to the introduction of hydrated aluminum and sulfate species, which facilitate water diffusion into the polymer matrix and promote partial film disintegration. At higher recycled alum contents, the water solubility progressively decreased to 148% and 119% for 20 wt% and 30 wt% Recy. Al, respectively. This reduction is associated with stronger interfacial interactions between the CS/PVA matrix and alum particles, including hydrogen bonding and electrostatic interactions, which restrict polymer chain mobility and limit water penetration. Notably, the composite containing 50 wt% Recy. Al exhibited a markedly reduced water solubility of 32%, indicating the formation of a highly compact and reinforced structure that significantly suppresses polymer dissolution.

Thus, the moisture content and water solubility results demonstrate that recycled alum content plays a decisive role in regulating water–polymer interactions. At lower filler contents, the hydrophilic nature of recycled alum enhances water uptake, whereas at higher loadings, polymer–filler interactions and structural densification dominate, leading to improved water resistance of the biocomposite films.

### 3.8. Biological applications

#### 3.8.1. Antimicrobial sensitivity tests

The antimicrobial activities of pristine CS/PVA, recycled alum (Recy. Al), can waste, and CS/PVA biocomposites containing different filler loadings (5–50 wt%) was evaluated using the inhibition zone diameter method against Gram-negative bacteria (*E. coli*, *Proteus* sp.), Gram-positive bacteria (*S. aureus*, *B. subtilis*), and fungi (*S. cerevisiae*, *A. niger*) as depicted in Table 3. A clear correlation was observed between alum loading and the antimicrobial performance. The activity progressively increased from 5 wt% to 20 wt% Recy. Al, with CS/PVA–20 wt% composite exhibiting the strongest antimicrobial effect, producing inhibition zones ranging from 33–45 mm. The antimicrobial activities of CS/PVA-20 wt% Recy. Al nearly is nearly three times more than standard tested antibiotics used, which included Gentamycin (1 µg mL^−1^), Amoxicillin (2 µg mL^−1^) and Fluconazole (4 µg mL^−1^) (Oxoid – UK).

**Table 3:** Antimicrobial sensitivities tested compounds at 400 µg using inhibition zones diameter (mm), compared with standard antibiotics

Compounds	Inhibition zone diameter (mm)					
	Bacteria				Fungi	
	*E. coli*	*Proteus* sp*.*	*S. aureus*	*B. subtilis*	*S. crevice*	*A. niger*
Cans waste	Negative	Negative	Negative	Negative	Negative	Negative
Recy. Al	13 ± 1	12 ± 1	13 ± 1	10 ± 1	11 ± 1	9 ± 1
Pristine CS/PVA	13 ± 1^a^	9 ± 1^a^	12 ± 1^a^	10 ± 1^a^	12 ± 1^a^	9 ± 1^a^
CS/PVA-5 wt% Recy. Al	21 ± 2^b^	19 ± 2^b^	20 ± 2^b^	19 ± 2^b^	23 ± 2^b^	17 ± 2^b^
CS/PVA-10 wt% Recy. Al	36 ± 2^b^	29 ± 2^b^	34 ± 3^b^	28 ± 3^b^	35 ± 3^b^	30 ± 3^b^
CS/PVA-20 wt% Recy. Al	45 ± 3^b^	33 ± 3^b^	41 ± 4.0^b^	39 ± 3.0^b^	45 ± 3^b^	35 ± 3^b^
CS/PVA-30 wt% Recy. Al	16 ± 1^b^	15 ± 1^a^	15 ± 1^a^	12 ± 1^a^	13 ± 1^a^	14 ± 1^a^
CS/PVA-50 wt% Recy. Al	10 ± 1^a^	11 ± 1^a^	11 ± 1^a^	10 ± 1^a^	13 ± 1^a^	10 ± 1^a^
Gentamycin (1 µg mL^−1^)	**15 ± 1^a^**	**11 ± 1^a^**	—	—	—	—
Amoxicillin (2 µg mL^−1^)	—	—	**15 ± 1^a^**	**13 ± 1^a^**	—	—
Fluconazole (4 µg mL^−1^)	—	—	—	—	**14 ± 1^a^**	**16 ± 1^a^**

a The changes are insignificant at *p* ≤ 0.05.

b The changes are significant at *p* ≤ 0.01.

This enhancement is attributed to the synergistic interactions between the alum-derived ionic species and the protonated CS/PVA matrix, which collectively disrupts microbial cell membranes and interferes with metabolic pathways. These results are consistent with reports showing that metal salts and polysaccharide-based matrices exert antimicrobial effects through ion release, electrostatic interactions, and membrane destabilization.^62^ However, at higher loadings (≥30 wt%), antimicrobial efficacy declined, with the CS/PVA–50 wt% composite showing significantly lower inhibition zones (10–13 mm). This reduction is attributed to alum agglomeration, which decreases the surface accessibility of active sites and possibly limits the diffusion of alum ions through microbial membranes at very high filler contents.

The MIC and MBC assays further supported the antimicrobial trends. The composites containing 10 wt% and 20 wt% Recy. Al demonstrated MBC/MIC tolerance ratios of 2, classifying them as bactericidal/fungicidal agents rather than merely growth-inhibiting (static) agents, following the criteria described by Mogana *et al.*^62^ A ratio ≤4 is typically indicative of cidal action, although this may vary depending on the microbial strain, environmental conditions, treatment duration, and agent concentration. These findings confirm that alum-containing CS/PVA biocomposites, particularly at 10–20 wt% Recy. Al, exert strong killing activity rather than simply suppressing microbial growth.

Statistical analysis showed significant differences in the antimicrobial activity of composites containing 5–20 wt% Recy. Al (*p* ≤ 0.01), confirming that the chemical and morphological modifications induced by alum incorporation play a crucial role in enhancing the biological performance. These results position the optimized composites (10–20 wt% Recy. Al) as promising antimicrobial materials for use in biomedical coatings, food packaging, wound dressings, and water purification systems.

#### 3.8.2. Evaluation of antimicrobial mode of action

The antimicrobial behavior of the composites was further analyzed using the minimum inhibitory concentration (MIC) and minimum bactericidal concentration (MBC) values were obtained (Table 4). These parameters determine whether the antimicrobial effect is cidal (kills microorganisms) or static (inhibits microbial growth). When the MBC/MIC ratio is ≤4, the antimicrobial action is considered cidal, whereas a ratio >4 typically indicates a static effect of the antimicrobial agent.^63^

**Table 4:** Minimum inhibitory concentrations (MICs), Minimum effective concentration of complete death (MCD), and tolerance values of biocomposites CS/PVA-(5–50 wt%) Recy. Al.^a^^,^^b^

Cpd.	^Bacteria^						Fungi					
	*E. coli*			*S. aureus*			*S. cerevisae*			*A. niger*		
	MICs (µg mL^−1^)	MCD (µg mL^−1^)	Tolerance value (MCD/MIC)	MICs (µg mL^−1^)	MCD (µg mL^−1^)	Tolerance value (MCD/MIC)	MICs (µg mL^−1^)	MCD (µg mL^−1^)	Tolerance value (MCD/MIC)	MICs (µg mL^−1^)	MCD (µg mL^−1^)	Tolerance value (MCD/MIC)
Recy. Al	125 ± 10	500 ± 40	4	125 ± 10	500 ± 45	4	250 ± 25	500 ± 55	4	125 ± 12	500 ± 45	4
Pristine CS/PVA	125 ± 10^c^	250 ± 23^d^	2	125 ± 10^c^	125 ± 14^d^	1	125 ± 10^d^	500 ± 55^c^	4	250 ± 12^c^	500 ± 45^c^	4
CS/PVA-5 wt% Recy. Al	62.5 ± 7^d^	125 ± 12^d^	2	62.5 ± 7^d^	125 ± 14^d^	2	62.5 ± 6^d^	125 ± 14^d^	2	125 ± 10^c^	500 ± 45^c^	4
CS/PVA-10 wt% Recy. Al	31.25 ± 4^d^	62.5 ± 4^d^	2	31.25 ± 4^d^	62.5 ± 8^d^	2	31.25 ± 3^d^	62.5 ± 6^d^	2	31.25 ± 4^d^	62.5 ± 4^d^	2
CS/PVA-20 wt% Recy. Al	7.8 ± 1^d^	15.626 ± 1^d^	2	7.8 ± 1^d^	15.426 ± 1^d^	2	62.5 ± 6^d^	125 ± 10^d^	2	62.5 ± 6^d^	125 ± 10^d^	2
CS/PVA-30 wt% Recy. Al	62.5 ± 6^d^	250 ± 30^d^	4	125 ± 12^c^	500 ± 49^c^	4	125 ± 12^d^	500 ± 50^c^	4	125 ± 10^c^	500 ± 35^c^	4
CS/PVA-50 wt% Recy. Al	125 ± 10^c^	500 ± 38^c^	4	125 ± 12^c^	500 ± 48^c^	4	125 ± 12^d^	500 ± 50^c^	4	125 ± 12^c^	500 ± 46^c^	4

a Bacteriocidal or fungicidal: tolerance value (MBC/MIC) was ≤4.

b Bacteriostatic or fungistatic: tolerance value (MBC/MIC) was >4.

c The changes are insignificant at *p* ≤ 0.05.

d The changes are significant at *p* ≤ 0.01.

The CS/PVA–10 wt% and CS/PVA–20 wt% Recy. Al composites showed MBC/MIC ratios of 2, confirming strong biocidal activity against both bacteria and fungi. This behavior arises from the integrated mechanisms of alum ion release, protonated CS/PVA interactions, and membrane destabilization. These biocidal composites demonstrate potential for real-world applications requiring rapid microbial eradication, such as disinfection materials, antimicrobial coatings, and biodegradable packaging.

#### 3.8.3. Cytotoxicity assay using *Artemia nauplii*

**Fig. 13:**
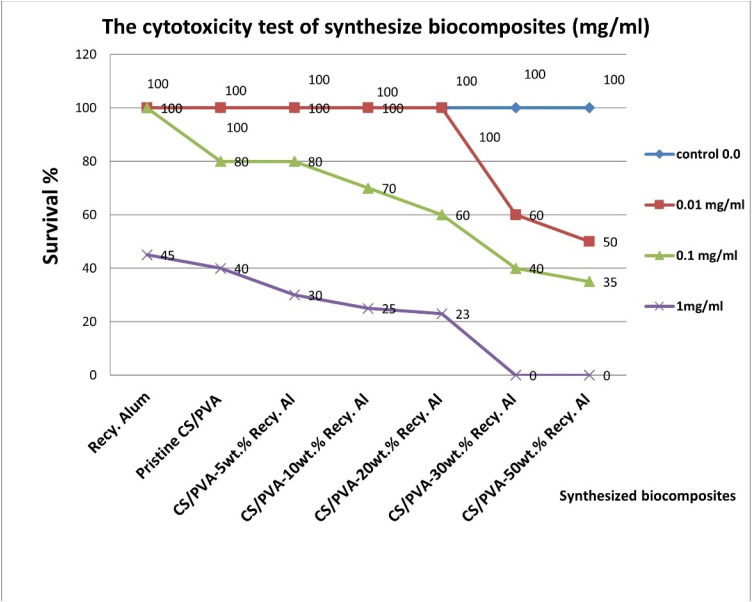
Cytotoxicity test of synthesized antimicrobial compounds (mg mL^−1^) using artemia in seawater.

The cytotoxicities of pristine CS/PVA, Recy. Al, and Recy. Al-filled composites (5–50 wt%) was evaluated using freshly hatched *Artemia* nauplii at concentrations of 0.0, 0.01, 0.1, and 1.0 mg mL^−1^ (Table 5, Fig. 13). After 24 h, the survival rates varied according to the alum content. At low and moderate Recy. Al loadings (≤20 wt%), the composites showed high nauplii survival (35–100% at 0.1 mg mL^−1^), indicating acceptable biocompatibility and minimal toxicity. These results demonstrate that moderate alum incorporation does not adversely affect the essential metabolic processes in aquatic organisms, consistent with the definitions of non-cytotoxic materials.^64^ Conversely, composites with high alum loadings (≥30 wt%), exhibited significantly reduced survival, including 0% survival at 1.0 mg mL^−1^ for the 30 wt% and 50 wt% samples. This behavior is likely attributed to the increased leaching of ionic species and higher surface activity, which may disrupt biological membranes or metabolic functions at excessive concentrations. These findings underscore the importance of optimizing alum content to balance antimicrobial activity and biocompatibility.

**Table 5:** The cytotoxicity test of synthesized antimicrobial compounds (mg mL^−1^) using artemia in seawater

Compounds	Survival %			
Concentration (mg mL^−1^)	0	0.01	0.1	1.00
Recy. Al	100	100	100	45
Pristine CS/PVA	100	100	80	40
CS/PVA-5 wt% Recy. Al	100	100	80	30
CS/PVA-10 wt% Recy. Al	100	100	70	25
CS/PVA-20 wt% Recy. Al	100	100	60	23
CS/PVA-30 wt% Recy. Al	100	60	40	0
CS/PVA-50 wt% Recy. Al	100	50	35	0

## 4. Conclusion

A new series of sustainable CS/PVA biocomposites was successfully developed by incorporating recycled potassium alum (Recy. Al) derived from aluminum can waste. The addition of alum notably improved both the structural integrity and functional performance of the CS/PVA matrix. The optimum formulation (30 wt% Recy. Al) exhibited a ∼55% increase in tensile strength and a 2.5-fold rise in Young's modulus compared to the pristine film. Thermal analysis revealed a distinct improvement in thermal stability, with *T*_d5_ and *T*_d10_ values increasing progressively from 188 °C and 239 °C (pristine) to 223 °C and 240 °C (50 wt% Recy. Al), along with an increase in char yield from 27% to 38%. The enhancement in stability is attributed to the presence of inorganic alum domains that restrict polymer chain mobility, facilitate char formation, and act as effective thermal barriers during degradation. XRD patterns suggested improved molecular organization, while SEM and EDS mapping confirmed homogeneous alum distribution and good interfacial compatibility within the polymer matrix. The composites retained high optical transparency and surface uniformity, reflecting strong polymer–filler compatibility. Future studies will include the evaluation of hardness and impact strength to further assess the mechanical durability of the developed biocomposites.

Importantly, all alum-reinforced CS/PVA composites exhibited broad-spectrum antimicrobial activity against both Gram-positive and Gram-negative bacteria, verifying their suitability for hygienic, biomedical, and eco-friendly applications. Their demonstrated antimicrobial cidal effect against common pathogens further underscores their potential in medical devices, food packaging, and water purification systems. Future perspectives include performing biodegradation studies to assess the environmental fate of these biocomposites and evaluate their long-term stability and real-world application performance.

## Author contributions

Mahmoud A. Abdelkawy: conceptualization, methodology, validation, investigation, formal analysis, writing-original draft, writing – review & editing. Abdelbaset Shoker: investigation, methodology, validation, review & editing, and supervision. Badr Isamil Badr: antimicrobial investigation, data curation, validation, review & editing. Yehia A.-G. mahmoud: microbiological analysis, supervision, and review & editing.

## Conflicts of interest

The authors declare no competing interests.

## Data Availability

The data that support the findings of this study are available within the article.
